# Far-Infrared Radiation Ameliorates the Cognitive Dysfunction in an Alzheimer’s Disease Transgenic Mouse via Modulating Jak-2/Stat3 and Nrf-2/HO-1 Pathways

**DOI:** 10.1007/s12017-025-08860-2

**Published:** 2025-05-15

**Authors:** Wen Yang, Qiuxia Yu, Nick Wang, Koon Kit Lam, Zhi-Xiu Lin, Yan-Fang Xian

**Affiliations:** 1 School of Chinese Medicine, The Chinese University of Hong Kong, Shatin, N.T., Hong Kong SAR China; 2 Nick Wang Technology Limited, TML Tower, 3 Hoi Shing Road, Tsuen Wan, Kowloon, Hong Kong SAR People’s Republic of China; 3 Hong Kong Institute of Integrative Medicine, The Chinese University of Hong Kong, Shatin, Hong Kong SAR People’s Republic of China

**Keywords:** Far-infrared radiation, Alzheimer’s disease, TgCRND8 mice, Cognitive dysfunction, Jak-2/Stat3 pathway, Nrf-2/HO-1 pathway

## Abstract

Alzheimer’s disease (AD) is the primary cause of dementia in the elderly. However, effective therapies that modify the disease process in AD remain elusive. Far-infrared radiation (FIR) is commonly utilized as a complementary treatment a range of disease, for example insomnia and rheumatoid arthritis. In this research, we explored how FIR light impacts the cognitive functions of TgCRND8 AD mice and elucidated its underlying molecular mechanism. The cognitive capabilities of TgCRND8 mice assessed by employing the Morris water maze. The concentrations of IL-1β, TNF-α, IL-4, Aβ40, and Aβ42 protein were assessed by enzyme-linked immunosorbent assay. Immunostaining was conducted to assess the Aβ deposits and microglial presence in the brains of TgCRND8 mice. Western blot was applied to detect the protein expressions of tau phosphorylation, amyloid-β (Aβ) production, Jak-2/Stat3, and Nrf-2/HO-1 pathways. The results indicated that FIR light notably ameliorated the cognitive impairments of the AD mice, reduced both Aβ deposition and tau protein hyperphosphorylation at sites of Thr205, Ser369, Ser404, and Thr181, suppressed the release of TNF-α and IL-1β, attenuated the ratios of p-Jak-2/Jak-2 and p-Stat3/Stat3, while increased the protein levels of IL-4, Nrf-2, and HO-1 in the brains of TgCRND8 mice. These findings amply demonstrated that FIR light ameliorated cognitive deficits of TgCRND8 mice via reducing both Aβ burden and tau protein hyperphosphorylation, suppressing the neuroinflammation, and restoring the levels of the oxidative-related proteins through modulating Jak-2/Stat3 and Nrf-2/HO-1 pathways. These experimental findings indicate that FIR light treatment is a promising treatment approach for AD.

**Supplementary Information:**

The online version contains supplementary material available at 10.1007/s12017-025-08860-2.

## Introduction

Alzheimer’s disease (AD), a prevalent ongoing neurodegenerative condition among elderly people, characterizes with amnestic disorder such as loss of memories, and loss of learning and memory functions (Graff-Radford et al., 2021). The development of AD involves multiple factors, including abundant deposition of β-amyloid (Aβ), tau protein hyperphosphorylation that forms the senile plaques, oxidative stress and neuroinflammation, all of which collectively contribute to synaptic loss and neuronal death in the AD brains, leading to cognitive impairments (Ma et al., 2022). Currently, the available therapeutic agents for AD only provide symptomatic relief while they are unable to interrupt the progress and fatal outcome of AD. In addition, many of the medications caused unwanted side effects in AD patients, such as hypertensive crisis, nausea, vomiting, and diarrhea (Briggs et al., 2016). Moreover, despite tremendous efforts made in recent decades to develop effective anti-AD drugs, there has been persistent failure of Aβ- and tau-targeting therapeutic strategies in clinical trials. There clearly exists an urgent necessity to develop new drugs or other innovative therapeutic approaches to slow down the onset and development of AD with less side effects on patients.

Recently, infrared radiation therapy as a non-pharmacological and noninvasive treatment modality has been applied for a wide range of diseases. Infrared radiation is a form of electromagnetic energy that has wavelengths between 750 nm and 1000 μm, which are longer than the visible light. International Commission on Illumination (CIE) categorizes infrared light as three sub-divisions according to the wavelength: (1) near-infrared radiation (0.7–1.4 μm), (2) middle infrared radiation (1.4–3.0 μm), and (3) far-infrared (FIR) radiation (3.0–1000 μm). FIR is known for its strong penetration and radiation force which could transfer energy through thermal radiation. Therefore, it is readily absorbed by body and converted into energy (Vatansever & Hamblin, 2012). Studies have substantiated FIR is a precise spatiotemporal selective, non-invasive, contact-free treatment, which poses low systemic toxicity, and is easy to administer. It has been applied to various conditions, such as inflammatory, cardiovascular, peripheral vascular, and neurological conditions (Qin et al., 2024). However, due to lack of standard therapeutic parameters as well as the unclear of underlying mechanisms whether FIR treatment has a satisfactory therapeutic effect remains to be studied. Previous studies have reported that mid infrared light treatment could ameliorate the cognitive deficits of APP/PS1 mice, a transgenic AD mouse model, via regulating gut microbiota, and enhancing the microglial phagocytosis to clean Aβ deposition (Li et al., 2022; Wang et al., 2020). Drawing on these literatures, we hypothesized that FIR facilities, such as FIR light, could possess significant anti-AD efficacy on TgCRND8 transgenic AD mice. The testing and validation of this hypothesis and elucidation of the underlying molecular mechanisms would further advance our knowledge about the potential of FIR facilities as innovative anti-AD therapeutic strategies.

## Materials and Methods

### Animals

Male wild-type (WT) C57BL/6 mice and TgCRND8 (Tg) mice were sourced from the Laboratory Animal Services Centre (LASEC), The Chinese University of Hong Kong (CUHK) (Hong Kong, China). The mice were kept in a controlled environment with a 12-h light/12-h dark cycle and had unrestricted access to food and water. Experimental animal procedures in this study were received approval from the Animal Experimentation Ethics Committee of CUHK (Approval No.: 22-201-MIS).

### FIR Spectrum Emitting Device

An FIR spectrum transmitter from EFFIT LITE® (Nick Wang Technology Limited, Hong Kong, China) that reliably emits a FIR spectrum in the wavelength range of 4–20 μm, with an average photon emissivity exceeding 80% was applied in this research (Chen et al., 2022).

### FIR Light Treatment

**Fig. 1:**
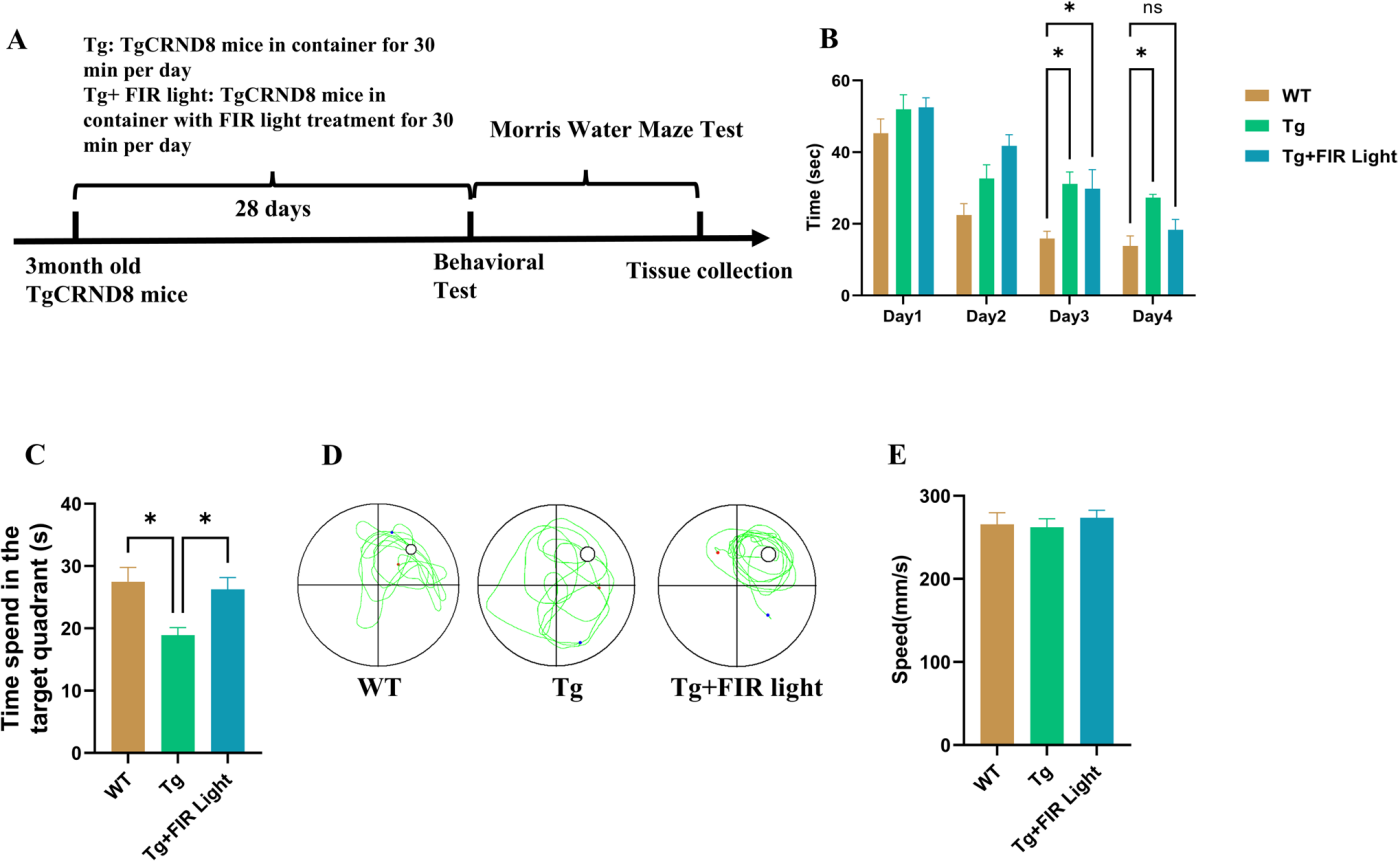
FIR light treatment ameliorated the learning and memory deficits of TgCRND8 mice. **A** Treatment schedule of FIR light on the TgCRND8 mice. **B** Escape latency of the mice to find the platform in training trial. **C** The time of the mice spent in the quadrant which contain the platform in the prob test. **D** Representative tracking routes of mice in the probe test. **E** The mean swimming speed of the mice in the prob test. Data were means ± SEM (*n* = 6). *p < 0.05; ns, not significant

At 3 months of age, TgCRND8 mice were indiscriminately assigned to the following groups: sham treatment group (Tg), the FIR light treatment group (Tg-FIR light treatment). WT (C57BL/6) mice at three months old were designed as normal control. Mice in Tg-FIR light treatment group were exposed to the FIR light in mouse restrainer (8.5 × 2.6 × 2.6 cm) for 30 min once daily, while the mice from Tg and WT groups were only kept in the mouse restrainer for 30 min once daily. For the FIR light treatment procedure, the FIR light was placed 1 cm above the mouse head directly, and the FIR light was turned on for 30 min. The treatment lasted for 28 consecutive days. The schedule of the animal experiment is shown in Fig. 1A.

### Morris Water Maze Test (MWMT)

After 28-day treatment with FIR light, MWMT was conducted on all mice to evaluate the cognitive function as detailed in our prior research (Yang et al., 2023). In summary, MWMT setup includes a 120 cm-diameter pool and four distinct symbols on the pool’s walls dividing the water surface into four quadrants. A camera positioned above the pool recorded the movement of the mice. The water temperature was kept at around 25 °C. This test comprised two main stages: the training trial and the spatial probe test.

Spatial trial was conducted to find the hidden platform in the water maze for four consecutive days. During the training trial, a platform (8 cm in diameter, 20 cm in height) was placed in the middle of the designated quadrant, submerged 1 cm below the water surface to keep it hidden from the mice. One day before the first trial, each mouse received 4 times of pre-training: mouse was put on the platform for 20 s, then given a 30-s free swim and then assisted to the platform where it was allowed to rest for another 20 s. On the first day, each mouse had 60 s of swimming time and 20 s of residence time on the platform. From the 2nd to 4th day, the platform residence time was reduced to 2 s. Each day, the mouse was introduced into the water from different directions three times, and the duration taken to locate the platform (escape latency) was recorded. If the mouse failed to find the target platform within 60 s, it was gently directed to the platform and its escape latency was recorded as 60 s.

In the spatial probe test, conducted 24 h after the training session, the platform was taken away. The mouse was put in the pool and monitored for 60 s. The time spent in the quadrant where the platform had been, along with the swimming trajectory, was recorded as indicators of spatial memory.

### Immunofluorescence

Immunofluorescence staining was conducted following the previous study with minor modifications (Li et al., 2019; Xu et al., 2023). Following the behavioral assessment, the mice were euthanized via cervical dislocation and subsequently perfused with 0.9% saline. The brain tissue were extracted and immersed in 4% paraformaldehyde solution (PFA) for 24 h. The brains were sequentially soaked in 10%, 20%, and 30% sucrose solutions to remove water, embedded in Tissue-Tek OCT compound, and stored at -80 °C. Coronal sections of the brain, each 8 μm thick, were prepared using a cryostat and rinsed in PBS.

When conducting staining, the slides were incubated with 5% BSA and 5% donkey serum at room temperature for 60 min for blocking the non-specific signal. The slides were then place at 4 °C with primary antibodies overnight, including Aβ antibody (1:1000, A5213, Sigma) and ionized calcium binding adapter molecule 1 (Iba1) (1:1000, ab178846, Abcam). After being rinsed with PBS for three times, the fluorescent secondary antibodies: IgG H&L (Alexa Fluor® 488 and Alexa Fluor® 647) were reactive with the sections at room temperature for 1 h. In order to stain the nuclei, the sections were treated with NucBlue™ Fixed Cell ReadyProbes™ Reagent (DAPI) (Cat No.: R37606, Invitrogen) for 10 min, and then rinsed with PBS for 3 times. The stained sections were mounted with ProLong™ Diamond Antifade Mountant (Cat No.: P36965, Invitrogen). Images were acquired by a ZEISS microscope (ZEISS Imager A2, Germany).

### Enzyme-Linked Immunosorbent Assay (ELISA)

The protein levels of interleukin (IL)-1β, TNF-α, IL-4, Aβ_40_ and Aβ_42_ in the cerebral cortex were determined using ELISA kits (Cat No.: ab197742 for IL-1β, Abcam; Cat No.: ab108910 for TNF-α, Abcam; Cat No.: ab100710 for IL-4, Abcam; Cat No.: KMB3481 for Aβ_40_, Invitrogen; Cat No.: KMB3441 for Aβ_42_, Invitrogen) following the manufacturer’s instruction. The final levels of TNF-α, IL-1β, IL-4, Aβ_40_, and Aβ_42_ were then adjusted according to its protein content, which was measured by a BCA protein assay kit (Cat No.: 23225, Invitrogen).

### Western Blot Analysis

The levels of specific molecules involved in Aβ production and tau protein phosphorylation in the cerebral cortex and hippocampus of TgCRND8 mice were assessed using Western blotting following a modified version of the previous method (Xu et al., 2023).

Tissue samples were homogenized and extracted using radioimmunoprecipitation assay (RIPA) lysis buffer, supplemented with a protease inhibitor cocktail. The homogenates were centrifuged at 12,000 g for 15 min at 4 °C, and the supernatant was gathered for analysis. Samples were then separated using SDS-PAGE gels and transferred to polyvinylidene fluoride (PVDF) membranes. The membranes were incubated for 60 min with 5% nonfat milk at room temperature for blocking and subsequently incubated overnight at 4 °C with various primary antibodies, including: ADAM metallopeptidase domain 10 (1:500, sc-48400, Santa Cruz), amyloid precursor protein (APP) (Thr688) (1:1000, 6986S, Cell Signaling Technology), beta-site-APP cleaving enzyme 1 (BACE1) (1:5000, ab108394, Abcam), Heme oxygenase 1 (HO-1) (1:1000, 26416S, Cell Signaling Technology), insulin degrading enzyme (IDE) (1:500, sc-393887, Santa Cruz), Janus kinase 2 (Jak-2) (1:1000, 3230S, Cell Signaling Technology), NFE2 like BZIP transcription factor 2 (Nrf-2) (1:2000, 16396–1-AP, Proteintech), p-Jak-2 (1:1000, 3776S, Cell Signaling Technology), p-Tau (Thr181, Thr205, Ser396, Ser404) (1:1000, 12885S, Cell Signaling Technology; ab254410, ab109390, ab92676, Abcam), p-Stat3 (1:1000, 9145S, Cell Signaling Technology), Signal transducer and activator of transcription 3 (Stat3) (1:1000, 12640S, Cell Signaling Technology), Tau 5 (1:500, sc-58860, Santa Cruz), and GAPDH (1:5000, ab8245, Abcam). After rinsing in Tris-buffered saline containing Tween 20, the membranes were treated with suitable secondary antibodies (1:3000, Cell Signaling Technology) and incubated for one hour at room temperature. Protein bands were visualized using SuperSignal™ Western Blot Enhancer (Cat No: 46640, Thermo Scientific). The intensity of the protein bands was normalized to the intensity of GAPDH bands from the same samples and quantified using Image J (Xu et al., 2023; Yang et al., 2023).

### Statistical Analysis

All data were presented as means ± standard error of the mean (SEM). Statistical analyses were conducted using GraphPad Prism version 8.0. Significant differences were assessed using an unpaired t-test or one-way/two-way analysis of variance (ANOVA) followed by Tukey’s or Dunnett’s multiple comparison tests. A p-value of below 0.05 was deemed statistically significant.

## Results

### FIR Light Treatment Ameliorated the Learning and Memory Deficit in TgCRND8 Mice

MWMT is commonly utilized to assess the spatial memory abilities of animals, leveraging their natural instinct to seek safety and their spatial orientation skills to escape the water (Lissner et al., 2021). This test is particularly effective in highlighting dysfunction in the hippocampus. Our results showed that the WT mice located the platform more quickly than TgCRND8 mice during the training trials (Fig. 1B). However, the learning and memory deficits in the TgCRND8 mice was enhanced by the treatment of FIR light (*F* (2, 77) = 4.81, *p* < 0.001). On day 4 of the training session, the FIR light-treated TgCRND8 mice exhibited escape latencies similar to those of the WT mice. In the spatial probe test, compared to the TgCRND8 mice without treatment, the FIR light-treated TgCRND8 mice spent more time in the target quadrant (*p* < 0.05) (Fig. 1C, D). Notably, swimming speed of TgCRND8 mice had no difference among different groups including the WT group, TgCRND8 mice vehicle control group, and FIR light treatment group (Fig. 1E). These results amply demonstrated that FIR light treatment effectively improved the cognitive deficit of TgCRND8 mice.

### FIR Light Reduced Aβ Plaques in the Brains of TgCRND8 Mice

**Fig. 2:**
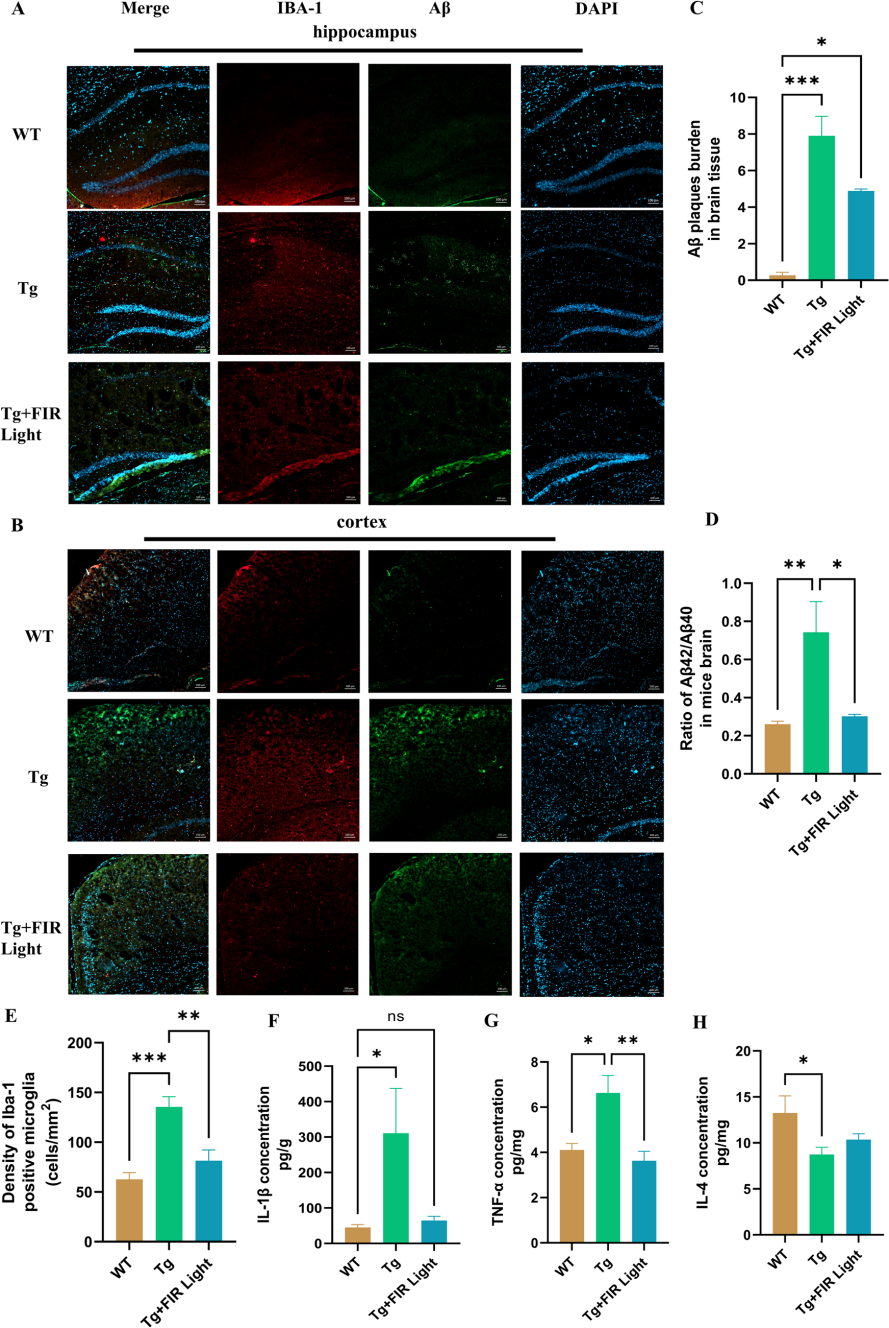
FIR light treatment ameliorated Aβ deposition and cytokine expression in the cortex and the hippocampus of the brains. **A**, **B** Representative images of Aβ staining in the cerebral cortex and the hippocampus from the WT, TgCRND8 and FIR light-treated TgCRND8 mice. **C** Aβ plaque density in the cerebral cortex and the hippocampus of the mice (*n* = 3). **D** The ratio of levels of Aβ_42_/Aβ_40_ of the cerebral cortex of the mice (*n* = 6). **E** Density of Iba-1 positive microglia in the cerebral cortex and the hippocampus of the mice (*n* = 3). **F–H** The levels of IL-1β, TNF-α, and IL-4 of the cerebral cortex of the mice (*n* = 6). Data were means ± SEM. *p < 0.05, **p < 0.01 and ***p < 0.001. *ns* not significant

Aβ pathology is a key characteristic of AD, and previous studies indicating that Aβ deposition in the brain would lead to subsequent pathological events in the AD progression, such as neuronal death and cognitive dysfunction (Frisoni et al., 2022). We aimed to define whether FIR light could help in alleviation of Aβ levels in the brains of AD mice. Compared to the WT mice, TgCRND8 mice exhibited abundant Aβ plaques in the brain’s cognitive region, including cerebral cortex and hippocampus (Fig. 2A-B). A distinguished decrease of Aβ plaques in these brain regions of the FIR light-treated mice was observed when compared to the TgCRND8 mice (Fig. 2C). We then measured the two primary Aβ peptides that precede plaque formation. The ratio of Aβ_42_/Aβ_40_ in cerebral cortex was significantly reduced following the FIR light treatment (Fig. 2D). These results demonstrated that FIR light notably reduced Aβ burden in the brains of TgCRND8 mice.

### FIR Light Suppressed Neuroinflammation in the Brains of TgCRND8 Mice

Neuroinflammation has been reported to participate in both the initiation and progression of AD. This process is primarily driven by key glial cells in the central nervous system (CNS), such as astrocytes and microglia which are responsible for initiating neuroinflammation (Deng et al., 2023). Accordingly, contrasted with the untreated TgCRND8 mice, FIR treatment markedly suppressed the density of Iba-1 positive microglia in cortex and hippocampus of TgCRND8 mice (*p* < 0.05) (Fig. 2A, B, and E). The release levels of cytokines such as IL-1β and TNF-α were notably lower in cerebral cortex following the FIR light treatment (Fig. 2F and G), while IL-4 in the cortex was not increased in FIR light-treated group (Fig. 2H).

### FIR Light Inhibited the Aβ Production by Modulating the APP Processing in TgCRND8 Mice

**Fig. 3:**
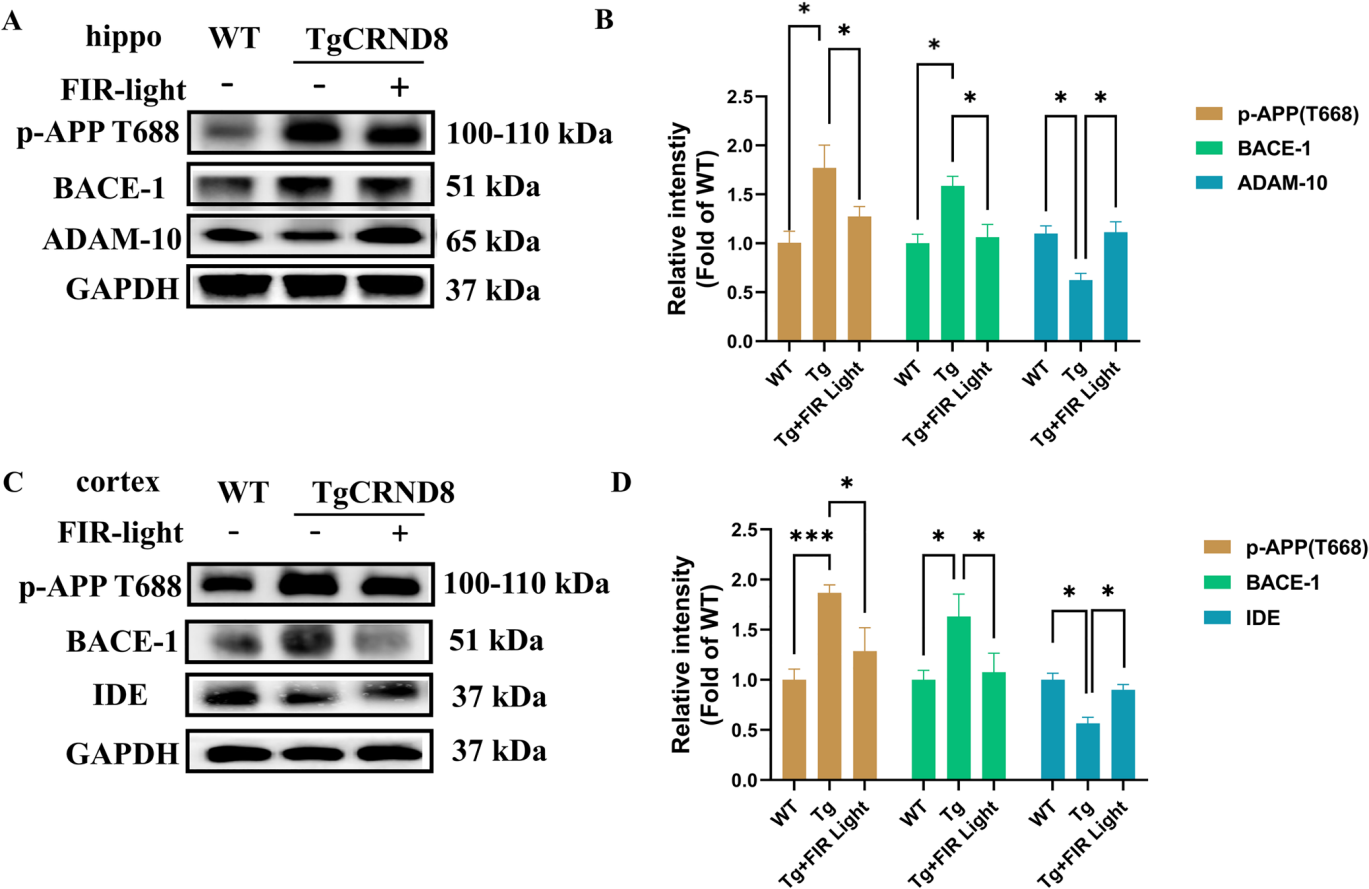
FIR light treatment modulated Aβ production and clearance in the cortex and the hippocampus of the brains. **A** The Western blot analysis of key molecules involved in the process of Aβ production in hippocampus of the mice. **B** Expression levels of p-APP, BACE-1 and ADAM-10 in the hippocampus of the mice. **C** The Western blot analysis of the key molecules involved in the process of Aβ production in the hippocampus of the mice. **D** Expression levels of p-APP, BACE-1 and IDE-1 in the cortex of the mice. Data were means ± SEM, *p < 0.05, **p < 0.01, and ***p < 0.001

The Aβ accumulation and aggregation are primarily result from an imbalance between Aβ production and clearance (Liu et al., 2016). According to the results of immunofluorescent staining and ELISA, FIR light treatment effectively reduced the Aβ burden in the brains of TgCRND8 mice. We next studied the effects of FIR light on the key molecules in Aβ production and clearance. The Western blot results indicated that FIR light could significantly reduce the protein expression of APP processing secretases, including β-secretase p-APP T688 and BACE-1, in both the cortex and hippocampus of TgCRND8 mice, while increase the expression of α-secretase ADAM-10 in the hippocampus and Aβ-degrading protease IDE in the cortex (Fig. 3A–D). We conclude that FIR light decreased Aβ burden through inhibiting Aβ production as well as increasing Aβ clearance pathway.

### FIR Light Inhibited the Tau Phosphorylation in the Brains of TgCRND8 Mice

**Fig. 4:**
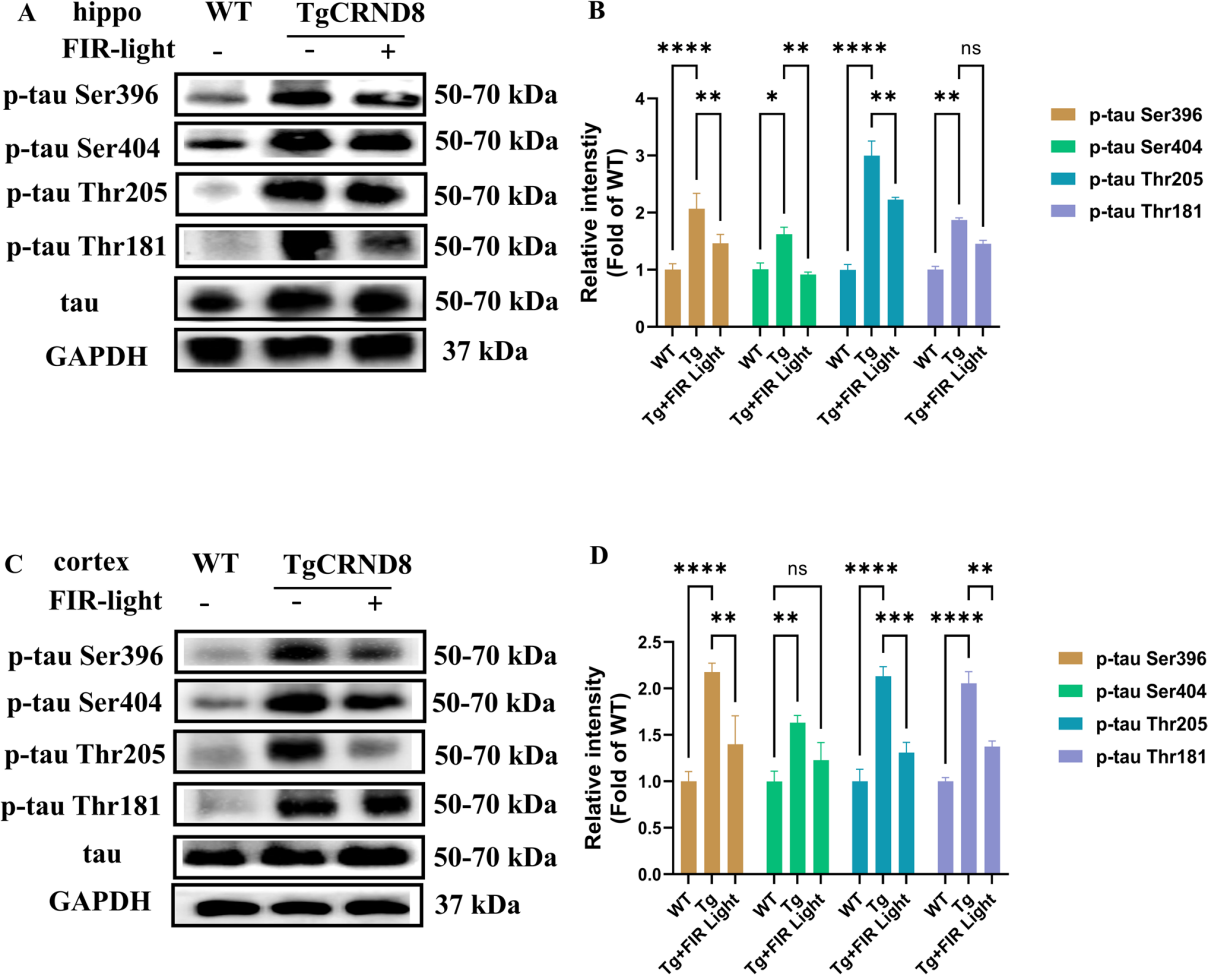
FIR light treatment reduced the phosphorylation of tau protein in the cortex and the hippocampus. **A** The Western blot analysis of phosphorylated tau protein in the hippocampus of the mice. **B** Expression levels of p-tau Thr205, p-tau Ser369, and p-tau Ser404 and p-tau Thr181 in the hippocampus of the mice. **C** The Western blot analysis of phosphorylated tau protein in cortex of the mice. **D** Expression levels of p-tau (Thr205), p-tau (Ser369), and p-tau (Ser404) and p-tau (Thr181) in the cortex of the mice. Data were means ± SEM, *p < 0.05, **p < 0.01 and ****p < 0.0001. *ns* not significant

Tau pathology is another key characteristic of AD. Tau protein hyperphosphorylation contributes to the development of neurofibrillary tangles (NFTs) and augments the progression of AD (Chu & Liu, 2019). In earlier research, we have found that the tau phosphorylation is increased in the TgCRND8 mice brains (Qu et al., 2023). The Western blot results demonstrated that FIR light could significantly inhibit the expression levels of phosphorylated tau proteins in two sites, including p-tau(Thr205) and p-tau(Ser369) both in the cortex and hippocampus, as well as reduced the expression of p-tau(Ser404) in the hippocampus and the level of p-tau(Thr181) in the cortex of the TgCRND8 mice (Fig. 4A–D). Accordingly, we speculated that FIR light could reduce the phosphorylation of tau protein at different sites in the brains of the TgCRND8 mice.

### FIR Light Inhibited the Jak2/Stat3 Signaling and Modulated Nrf-2/HO-1 in TgCRND8 Mice

**Fig. 5:**
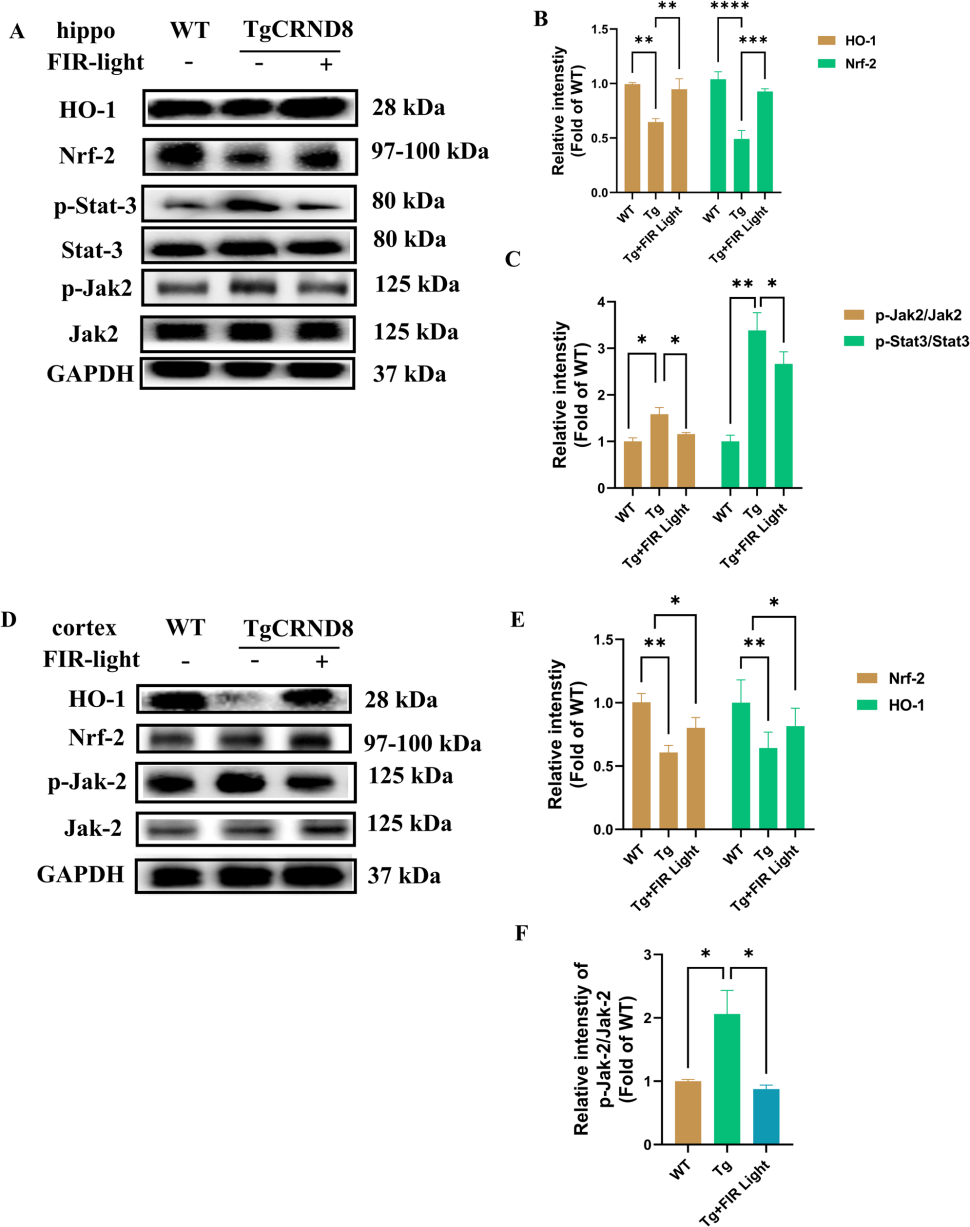
FIR light treatment modulated the expression of Jak-2/Stat3 and Nrf-2/HO-1 signaling in the TgCRND8 mice. **A** The Western blot analysis of Jak-2/Stat3 and Nrf-2/HO-1 in the hippocampus of the mice. **B** Expression levels of Nrf-2, HO-1 in the hippocampus of the mice. **C** Expression levels of p-Jak2/Jak2, p-Stat3/Stat3 in the hippocampus of the mice. **D** The Western blot analysis of Jak-2 and Nrf-2/HO-1 in the cortex of the mice. **E** Expression levels of Nrf-2, HO-1 in the cortex of the mice. **F** Expression levels of p-Jak2/Jak2 in the cortex of the mice. Data were means ± SEM. *p < 0.05, **p < 0.01 and ****p < 0.0001

To further explore the impact of FIR light treatment on neuroinflammation, we evaluated the protein expression level of the Jak/Stat3 signaling pathway, which is known to regulate the neuroinflammatory processes and microglial polarization (Rusek et al., 2023). Moreover, Nrf-2/HO-1 signaling is known to be closely connect to oxidative stress and immune dysfunction (Osama et al., 2020). Western blot analysis revealed that FIR light could significantly suppress the ratio of p-Jak-2/Jak-2 both in the cortex and the hippocampus of TgCRND8 mice (Fig. 5A, C, D and F), and suppress the ratio of p-Stat3/Stat3 in the hippocampus of TgCRND8 mice (Fig. 5A and C). Levels of Nrf-2 and HO-1 were significantly reduced in TgCRND8, while FIR light could markedly increase the expression levels of Nrf-2 and HO-1 both in the cortex and the hippocampus of the TgCRND8 mice (Fig. 5A, B, D and E). We therefore speculated that FIR light could inhibit Jak-2/Stat3 signaling, while enhance Nrf-2/HO-1 signaling in the TgCRND8.

## Discussion

AD is a common neurodegenerative disease marked by a gradual deterioration in cognitive abilities (Xie et al., 2021). Accordingly, ameliorating cognitive impairment has been a target of AD treatment. FIR radiation specialty lamps and saunas have been developed to be safe, effective sources for generating therapeutic effects for many years in treating pain, arthritis, skin disease, and diabetic wound (Chen et al., 2021; Tsagkaris et al., 2022). There were also studies that evaluated the effects of FIR radiation treatment on AD. Recently study reported FIR light enhances cognitive functions in APP/PS1 mice via reducing Aβ plaque burden, decreasing neuroinflammation, and restoring the expression of the presynaptic protein synaptophysin, and gut microbiota dysbiosis (Li et al., 2022; Wang et al., 2020). However, in another study, it reported that FIR light fabric treatment for 5 months did not provide significant benefit on cognition and motor functions of 5 × FAD mice, suggesting that the exposure method and wavelength of FIR light need to be well-defined in order to get the effective results (Fukui et al., 2021). According to these studies, the limitation of FIR treatment has been showed that due to there is no standardized therapeutic parameters of FIR treatment. The treatment effect could be varied between different studies. In this study, we applied EFFIT LITE® as the FIR spectrum transmitter which stably radiates an FIR spectrum with a wavelength of 4–20 μm, and the device was put within 1 cm directly above the head of the 3-month-old TgCRND8 mice for 30 min exposure once every day. The results of MWMT showed the FIR light notably enhanced the cognitive function and spatial memory of the TgCRND8 mice after 28-days consecutive treatment. The findings amply suggested that FIR light could serve as a promising treatment for AD patients.

Aβ peptide-formed plaques, a key pathological feature of AD, lead to neuroinflammation and neuronal death. Therefore, reducing excessive Aβ in the brain is considered as a promising therapeutic approach for AD (Zhao et al., 2023). In the brains of individuals with AD, APP is cleaved by β- and γ-secretase into peptides vary from 39 to 43 amino acids depending on cleavage sites (e.g., Aβ_40_ or Aβ_42_) in the AD brain (Hampel et al., 2021). Besides, there are also mechanisms to expel Aβ from the brain such as IDE (Kurochkin et al., 2018). The imbalance between Aβ production and clearance leads to misfolding and aggregation of Aβ peptides, and extracellular accumulation of Aβ peptides, ultimately resulting in amyloid plaque formation (Jack et al., 2018). Our study indicated that FIR light treatment markedly decreased the ratio of Aβ_42_/Aβ_40_, the two primary Aβ peptides involved in Aβ plaque deposition, in the cortex of TgCRND8 mice. Moreover, FIR light suppressed the Aβ plaques both in the brain of the TgCRND8 mice as detected by the immunofluorescence staining when compared to the non-treatment group. Furthermore, FIR light treatment reduced the protein expression of BACE-1 (β-secretase) and p-APP (Thr688) which are related to Aβ production, while FIR light treatment could increase the expressions of ADAM-10 (α-secretase) and IDE (Aβ-degrading protease), the two enzymes responsible for clearance of Aβ. Taken together, our findings strongly suggested that the anti-AD property of FIR light treatment are partly attributed to its capacity to inhibit the Aβ production and accentuate its clearance.

NFTs, which is formed by the accumulation of tau aggregates, is another key pathological aspect of AD that is closely linked to, both spatially and temporally of neurodegeneration and the development of clinical symptoms (Ossenkoppele et al., 2022). Excessive phosphorylation of tau (p-tau) at different sites such as Thr181, Thr205, Ser396 and Ser404 increases the likelihood of tau forming insoluble paired helical filaments and NFTs (Barthélemy et al., 2019, 2020; Dujardin et al., 2020). In the previous study, enhanced activity of p-tau was observed in the TgCRND8 mice from 2 months of age when compared with the WT mice (Qu et al., 2023). In this study, FIR light could significantly inhibit the expression level of phosphorylated tau protein at different sites, including Thr205, Ser369, Ser404, and Thr181 in the brains of the TgCRND8 mice. These findings suggested that the cognition-improving property of FIR light is partly related to the inhibition of hyperphosphorylation of tau protein.

Neuroinflammation, an important pathogenesis of AD, has been reported to provide a link between Aβ pathology and NFTs (Chen & Yu, 2023; Kinney et al., 2018). In the CNS, microglia are immune cells that are sustained activated in AD and contribute to the continuous release of inflammatory factors that exacerbate Aβ accumulation and tau propagation (Leng & Edison, 2021). It has also been observed that inflammatory cytokines such as IL-1β and TNF-α are increased, while neuroprotective cytokines such as IL-4 and IL-10 are suppressed in the brains of AD patients (Kwon & Koh, 2020). In this study, immunofluorescence staining showed significantly increased densities of Iba-1-positive microglia in the brain of the TgCRND8 mice, while in the FIR light-treated group the activated microglia were reduced. Moreover, FIR light treatment decreased the levels of the pro-inflammatory cytokines such as IL-1β and TNF-α, while the decreased IL-4 was not affected by FIR light treatment.

Furthermore, Jak2/Stat3 pathway has been reported to be activated by cytokines, and plays a crucial role in AD development. When exposed to different cytokines, Jak2 gets phosphorylated at tyrosine residues, functioning as cell cycle regulator that protects cells from oxidative stress (Panda et al., 2024). In our previous study, the elevated level of p-STAT3/STAT3 was observed in the brains of the TgCRND8 mice, when compared with the WT mice (Qu et al., 2023). The results indicated that FIR light treatment reduced the ratios of p-Jak2/Jak2 and p-Stat3/ Stat3. The Nrf-2/ HO-1 signaling, a key endogenous antioxidant system, helps mitigate oxidative stress and enhances the expression of various endogenous genes (Huang et al., 2015). Animal studies have demonstrated that both Nrf-2 and HO-1 exhibit anti-inflammatory properties and inhibit the release of inflammatory factors such as TNF-α, IL-1β, and MIP-1, and they play a significant role in combating oxidative stress (Kim et al., 2022; Xue et al., 2019; Zhao et al., 2022). FIR light could also increase the expression levels of Nrf-2 and HO-1 both in the cortex and the hippocampus in TgCRND8 mice. Taken together, the results strongly indicated that the anti-AD effects of FIR light is link to its anti-neuroinflammatory property.

The Nrf2/HO-1 and JAK/STAT3 signaling pathways are closely related to oxidative stress and neuroinflammation, which are two key pathogenic mechanisms involved in AD. Oxidative stress and neuroinflammation are interrelated, amplifying each other’s effects in the progression of AD. Amyloid pathology and tauopathy are not only known to impair mitochondrial function but also trigger neuroinflammation by activate microglial and astrocytes, ultimately leading to neuron apoptosis in AD brain (George et al., 2022). JAK2/STAT3 pathway has be reported to be activated by extra cytokines and reactive oxygen species under AD condition (Wen & Hu, 2024). Inhibition of JAK2/STAT3 pathway would suppress the neuroinflammation, while enhancing antioxidant Nrf2/HO-1 pathway (Hindam et al., 2020). Recent studies have recognized Nrf2 as a key upstream regulator of cytokine production, laying the groundwork for Nrf2-mediated anti-inflammatory strategies relevant to neuroinflammation in AD (Kobayashi et al., 2016). The activation of HO-1 during inflammatory conditions may serve as an adaptive response to reduce cytotoxicity through various mechanisms. In this study, we observed that FIR treatment significantly reduced AD markers in TgCRND8 mice, including Aβ deposition in the brain and hypophosphorylation of tau protein. Our findings suggest that the activation of antioxidant pathways and the suppression of neuroinflammatory pathways are key mechanisms underlying the therapeutic effects of FIR treatment in AD.

## Conclusion

In summary, treatment with the FIR light at the wavelengths of 4–20 μm for 28 days could effectively ameliorate the cognitive dysfunction in the TgCRND8 mice, and the underlying molecular mechanisms involve the suppression of the Aβ deposition, hyperphosphorylation of tau, and neuroinflammation through modulating Jak-2/Stat3 and Nrf-2/HO-1 pathways. Our current experimental findings amply indicate that FIR light is a potential non-pharmacological therapy for AD. Clinical trial on the effectiveness and safety of FIR light on AD patients is warranted.

## Supplementary Information

Below is the link to the electronic supplementary material.

Supplementary file1 (PDF 354 KB)

## Data Availability

No datasets were generated or analysed during the current study.

## Abbreviations

Aβ: β-Amyloid
AD: Alzheimer’s disease
APP: Amyloid precursor protein
BACE1: Beta-site-APP cleaving enzyme 1
CIE: Commission on Illumination
CNS: Central nervous system
ELISA: Enzyme-linked immunosorbent assay
FIR: Far-infrared
GAPDH: Glyceraldehyde 3-phosphate dehydrogenase
HO-1: Heme oxygenase 1
IDE: Insulin degrading enzyme
MWMT: Morris water maze test
NFTs: Neurofibrillary tangles
PBS: Phosphate buffered saline
RIPA: Radio immunoprecipitation assay
SEM: Standard error of the mean
