# Supplementary figures and images for: Far-Infrared Radiation Ameliorates the Cognitive Dysfunction in an Alzheimer’s Disease Transgenic Mouse via Modulating Jak-2/Stat3 and Nrf-2/HO-1 Pathways
Source: Neuromolecular Med. 2025 May 15;27(1):34. doi: 10.1007/s12017-025-08860-2 (PMC12081534; doi:10.1007/s12017-025-08860-2)

Fig. 3A

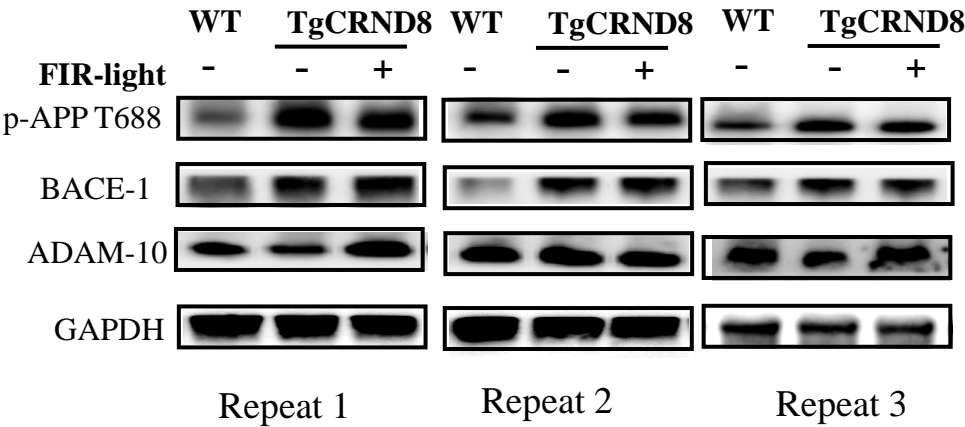

Fig. 3C

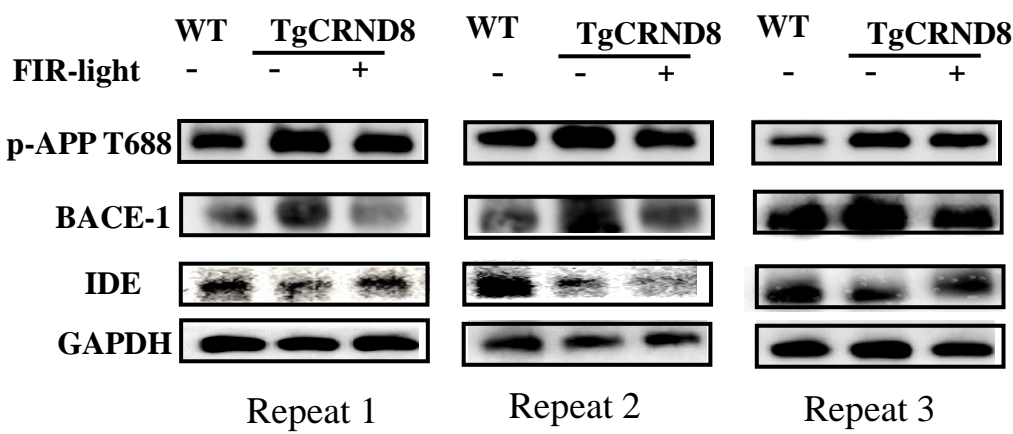

Fig. 4A

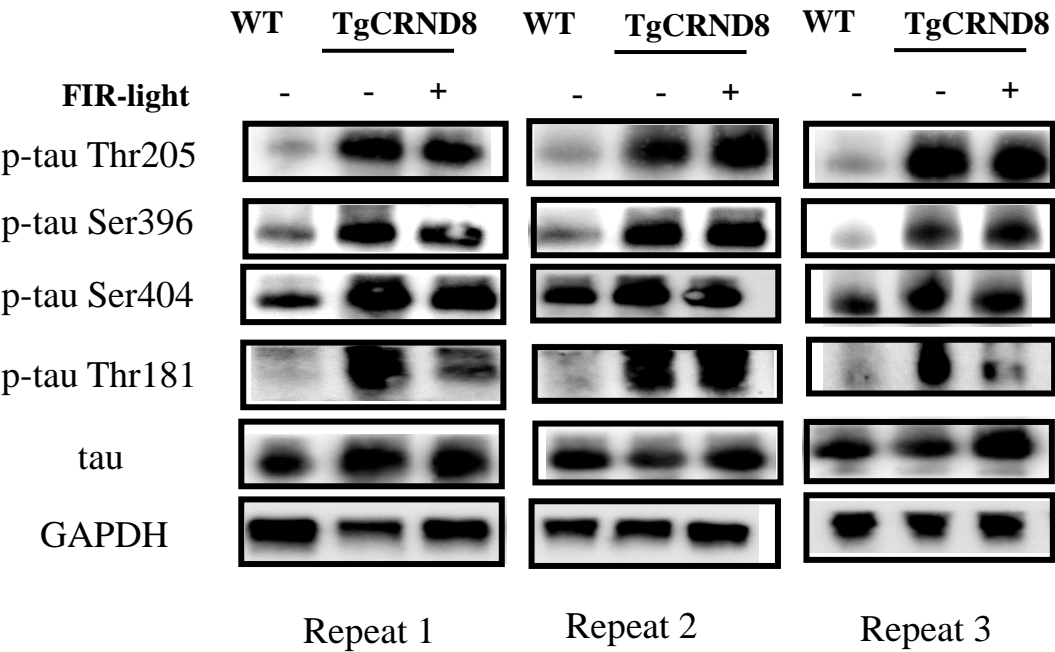

Fig. 4C

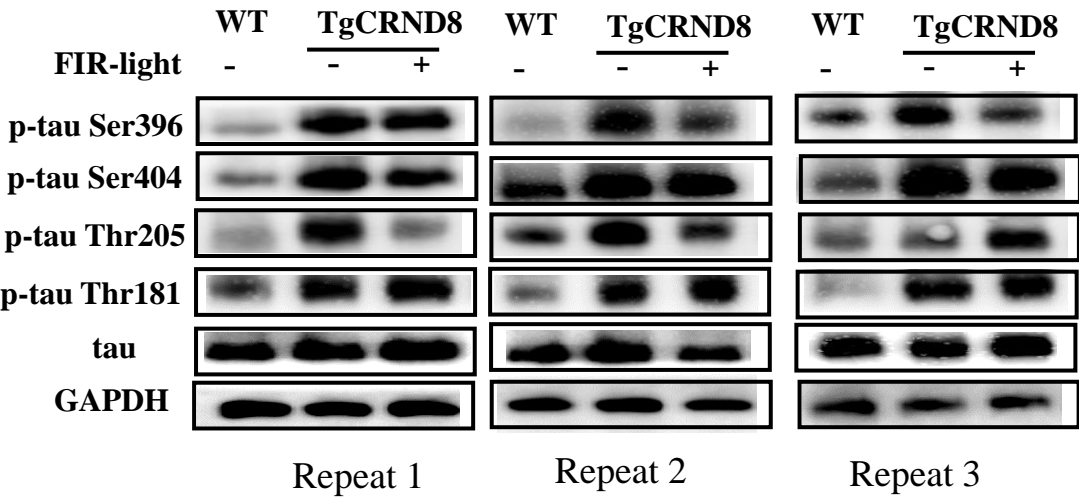

Fig.5A

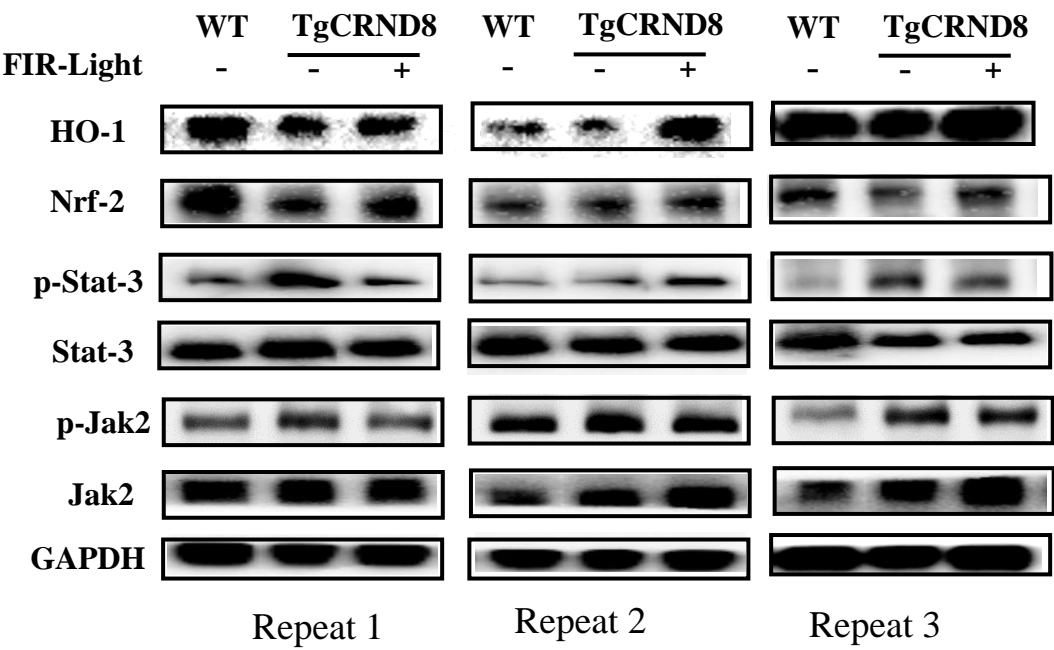

Fig.5D

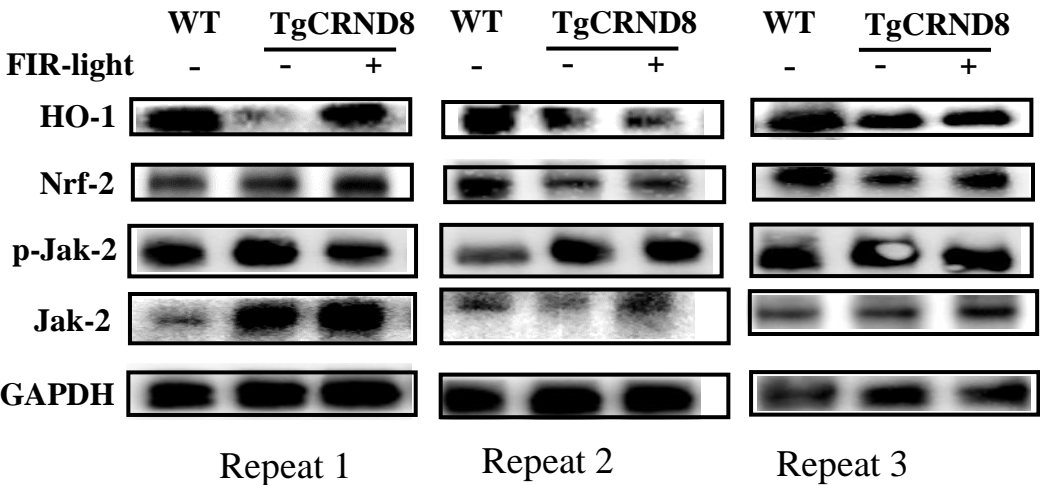

Supplement: Supplementary file 1 — Supplementary file1 (PDF 354 KB) [file 12017_2025_8860_MOESM1_ESM.pdf]
